# Patient Perspective in Saudi Arabia: What Qualities Make a Good and Competent Psychiatrist?

**DOI:** 10.7759/cureus.33022

**Published:** 2022-12-27

**Authors:** Norah F AlMuhanna, Omar T Sodagar, Omar O Al-Hayek, Feras A AlQahtani, Mahmoud A Alghomgham

**Affiliations:** 1 Psychiatry, Imam Abdulrahman Bin Faisal University, Khobar, SAU

**Keywords:** saudi arabia, patients’ perspective, competent, qualities, psychiatrist

## Abstract

Introduction

The good qualities of a psychiatrist can vary when asking a psychiatrist, a resident, a student, or even a patient. The patient’s perspective, however, is of utmost importance and can be extremely unique. Our study aims to visualize and analyze the perspective of outpatients of the psychiatric department at King Fahad Hospital University on what qualities make a good and competent psychiatrist. As psychiatric medicine depends a lot on the psychological aspect, it yields high importance on studying how patients perceive and see their doctors in different regions of the world. For this reason, we conducted our study in Al-Khobar, Kingdom of Saudi Arabia (KSA).

Materials and methods

A self-structured questionnaire named “What Qualities Make a Good and Competent Psychiatrist?” was developed and used to achieve the study objectives. It encompasses six sections: patients’ personal data and demographics, patients’ medical data, psychiatrists’ personal traits, knowledge domain, social domain, and clinical setting domain.

Results

After data collection and analysis, our results showed the following. Patients did not have a generalized preference for personal traits or demographics; however, they preferred Saudi and Arabic-speaking physicians among others. Patients preferred their psychiatrists to be up to date with current research. The score per item is the lowest in the clinical domain, indicating that for the respondents, the clinical domain is the most important trait of a psychiatrist. Of the patients, 66.8% reported that appropriate grooming and clothing were crucial for a psychiatrist. Empathy and proper communication were also very important from the patients’ points of view. Social and knowledge domains are also of extreme importance in our region.

Discussion

In our study, there seems to be no preference toward a psychiatrist’s demographic traits with the exception of a Saudi nationality and being an Arabic speaker. The traits explored in this study are categorized into three domains: clinical setting domain, knowledge domain, and social domain. Of the three domains mentioned, the clinical setting domain was deemed the most important, followed by the social and knowledge domains.

In a study conducted in Singapore, the social characteristics of the psychiatrist, which represented care and sympathy, were the main concern of patients. A study conducted in the UK showed that the participants were less concerned with the organizational social aspects of the medical processes than the clinical management components. Although the overall requirement for a knowledgeable, skilled, and socially competent psychiatrist and a proper clinical setting is sought by most psychiatric patients in different cultures and countries, there are also significant differences in the priorities of such characteristics. This emphasizes how every region has its standards for this occupation.

Conclusion

The results of this study suggest a variety of patients’ and guardians’ perspectives in different cultures with the clinical setting being an integral part of the psychiatrist’s practice in Al-Khobar, KSA. The results showed that the clinical setting domain was the most critical domain, followed by social and knowledge domains. To improve patient experience and satisfaction, certain actions should be taken into consideration in our region.

## Introduction

The American Psychiatric Association defines psychiatry as “the branch of medicine focused on the diagnosis, treatment, and prevention of mental, emotional, and behavioral disorders.” A psychiatrist is a medical doctor who is trained in treating mental health and substance use disorders [1]. Psychiatry as a specialty is relatively new when compared to older specialties in medicine such as surgery. It was first recognized in the late 1800s in England as a specialty of medicine. Psychiatric patients before were called lunatics or insane. This created a stigma around the specialty among physicians, patients, patients’ families, and caregivers [2,3].

The stigma surrounding psychiatry has been declining over the past years but is still present around the world. For example, a paper from the UK showed that this stigma can delay patient care since patients and their families are naturally skeptical about the specialty and the treatment of such conditions [4,5]. Due to the stigma around the specialty, it is important for physicians and psychiatrists to understand what factors contribute to the stigma and what qualities and merits make a good psychiatrist. Since the patient and his family took the first step in asking for help, it is our role as healthcare providers to understand the factors that determine the patient’s willingness to continue following up [6].

Psychiatric disorders can be misunderstood by the public and poorly recognized. Depression, anxiety, substance use, and other common mental disorders not only affect the patient but also their family, friends, and coworkers [7]. Psychiatric disorders represent 20% of the global burden of disability [8]. Proper psychiatric care assures good quality of life for the patient and the many people surrounding him [9].

In 2015, a group of psychiatrists and psychologists in South Korea, as well as Australia, studied the qualities of a good psychiatrist through the means of a survey given to the patients. Patients in the study defined a good psychiatrist as an active listener and a good communicator and one who has respect for confidentiality and professionalism [6]. Another study in 2014 conducted in Singapore by a group of psychiatrists and psychologists studied the qualities of a good psychiatrist from the patient’s perspective and the psychiatrist. Psychiatrists focused on the merits of professionalism and social interaction, keeping in mind the guidelines of practice and knowing that they are accountable for their actions. Patients, on the other hand, focused more on the similarity of gender, race, culture, and religion between patients and psychiatrists. Allowing more time for the psychiatric consult and letting the patient speak freely are associated with better patient understanding and outcomes [10].

The difference in the patient’s and physician’s perspectives can be seen in other specialties as well. According to a systematic review conducted by the European Task Force on Patient Evaluations of General Practice Care (EUROPEP), the most important quality patients look for is how well a physician communicates and relates to the patient, followed by their competence [11]. In other words, those patients expect to be involved in their treatment plan and to be aware of the options. Not involving patients in the treatment plan can cause major dissatisfaction to them [12]. A questionnaire-based study conducted in Scotland showed that patients prioritize a physician who listens to their needs and avoids harming them [13]. Moreover, in a separate study that was conducted in the Netherlands, patients appreciated a consultation time that was suitable to their situation, whereas the physician was more focused on a different aspect such as the continuity of care [14].

The good qualities of a psychiatrist can vary when asking a psychiatrist, a resident, a student, or even a patient. The patient’s perspective, however, is of utmost importance and can be extremely unique due to gender, race, culture, religion, previous interactions, mental disorders, and many other factors. Since culture, religion, and race can influence the perspective of patients, this study is conducted in Saudi Arabia to see the difference in results published in different countries, hopefully applying these findings to improve the quality of care in the psychiatric setting [15].

## Materials and methods

Study design and setting

An exploratory study design was adopted to reveal the characteristics and traits of a good psychiatrist from the perspective of patients, family members, and caregivers. This study was conducted at King Fahad Hospital of the University (KFHU), Al-Khobar, Saudi Arabia, from May 18 to July 12, 2022.

Subjects

Sample Size and Technique

Using convenience sampling, 804 patients who satisfied the inclusion criteria were selected and administered the questionnaire named “What Qualities Make a Good and Competent Psychiatrist?” Of the 804 patients, 199 have responded and completed the questionnaire, demonstrating a 24.75% response rate.

Inclusion Criteria

All patients and guardians visiting the outpatient psychiatry department (OPD) of the King Fahad Hospital of the University (KFHU) who are 18 years of age and above and who are not classified as having severe psychiatric illness according to the Diagnostic and Statistical Manual of Mental Disorders (DSM-5) were considered as the population for this study.

Exclusion Criteria

Patients under 18 years of age, classified as having severe psychiatric illness according to DSM-5 or not under the care of the OPD of KFHU were excluded from this study.

IRB Approval

The Imam Abdulrahman Bin Faisal University Institutional Review Board issued approval number IRB-UGS-2021-01-393.

Variables

Independent Variables

Demographical characteristics of the patients and their family members, such as age, sex, gender, nationality, social status, educational qualification, and residence, were considered as the independent variables. Concerning the influence of the psychiatrist’s traits on the respondents’ overall perception, the psychiatrist’s traits, such as knowledge and social domain, were the independent variables.

Dependent Variables

The knowledge domain, social domain, and overall perception of the patients and their family members were considered the dependent variables. Concerning the influence of the psychiatrist’s traits on the respondents’ overall perception, the respondents’ overall perception was the dependent variable.

Materials

In the current literature, there is a lack of comprehensive questionnaires that cover all domains studied as well as cultural and religious differences. As a result, a self-structured questionnaire named “What Qualities Make a Good and Competent Psychiatrist?” was developed by combining different existing questionnaires from other studies and adding questions relevant to our population to achieve this study’s objectives.

This questionnaire encompassed six sections: patients’ personal data and demographics (nine items), patients’ medical data (five items), psychiatrists’ personal traits (five items), knowledge domain (five items), social domain (eight items), and clinical setting domain (five items).

The respondent’s level of agreement with items in the knowledge domain, social domain, and clinical setting domain was expressed on a five-point Likert scale (1: totally disagree, 2: disagree, 3: neutral, 4: agree, 5: totally agree).

Regarding reliability, the internal consistency of the three domains, i.e., knowledge domain, social domain, and clinical setting domain, was assessed using Cronbach’s alpha (Table 1).

**Table 1:** Test for internal consistency of the items with the knowledge domain, social domain, and clinical setting domain

Domain	Cronbach’s alpha	Cronbach’s alpha based on standardized items	Number of items
Knowledge	0.827	0.831	5
Social	0.827	0.831	5
Clinical	0.307	0.468	5

Procedures

The questionnaire was uploaded on QuestionPro. A QR code of the questionnaire was distributed to the psychiatry outpatient department (OPD). Patients, family members, or caregivers who have recently visited the psychiatry OPD were contacted. After a brief explanation, consent was obtained, and QR codes were provided with instructions via short message service (SMS) or WhatsApp. No confirmation of a response was elicited.

Data analysis

Descriptive statistics were used to describe the demographic and medical data of the patients and the psychiatrist’s personal traits, knowledge, and social domain. Before starting with the data analysis, data cleaning was performed, which involved the following steps. Firstly, nonessential rows at the top of the dataset were removed so that each column header can be used as variable names. Secondly, data from respondents who did not complete the interview were removed from the dataset so that the analysis can be done only on the completed set of interviews. Lastly, variable names were shortened to avoid errors during the analysis. Short variable names also help in conducting the analysis efficiently.

Three separate composite scores were calculated for the knowledge domain, social domain, and clinical setting domain. The calculation of the composite scores was done as follows. Firstly, options against each of the questions within the domain were expressed using a five-point Likert scale. Secondly, the composite scores in each of the domains were calculated for each of the respondents by adding the scores of their selected options for each of the questions in the domain. Finally, since there are an unequal number of items in each of the domains, composite scores were divided by the number of items in the domain to make them comparable.

The higher the score per item of a respondent on a domain, the lesser its perceived importance for a good psychiatrist. For example, a respondent with a score per item of one on the knowledge domain gives more importance to knowledge than a respondent who has a score per item of two on the knowledge domain. Thereafter, descriptive statistics for each of the variables in the dataset were calculated. For categorical variables, proportions and percentages were calculated; for continuous variables, means and standard deviations were calculated. Additionally, for the continuous variables, Kolmogorov-Smirnov tests for normality assumption were performed. Apart from the “age” variable, none of the variables were found to be satisfying the assumption of normal distribution (Table 2).

**Table 2:** Test for the assumption of normal distribution for continuous variable

	Kolmogorov-Smirnov		
	Statistic	Degrees of freedom	Significance
Age	0.072	199	0.014
Knowledge domain score per item	0.123	199	0.000
Social domain score per item	0.146	199	0.000
Clinical setting domain score per item	0.101	199	0.000

The internal consistency of the three domains, i.e., knowledge domain, social domain, and clinical setting domain, was assessed using Cronbach’s alpha. While internal consistency was found to be good for the knowledge and social domains of the questionnaire (Cronbach’s alpha of 0.827 and 0.932, respectively), the internal consistency of the clinical setting set of questions was poor (Cronbach’s alpha of 0.307). However, it was found that dropping the question “I prefer physical clinics over virtual clinics (i.e., through phone, Zoom, etc.)” from the analysis increases Cronbach’s alpha to 0.592, which is a good score for internal consistency (Table 1).

An inter-item correlation matrix was generated for each of the three domains, namely, the knowledge domain, the social domain, and the clinical setting domain (Tables 3-5).

**Table 3:** Inter-item correlation between items in the knowledge domain

	I highly give importance to the psychiatrist’s knowledge and skills.	I like to know the psychiatrist’s credentials and qualifications before meeting them.	I look into a doctor’s subspecialty before choosing them.	I want my psychiatrist to be up to date on the most recent research available.	The most important trait in a psychiatrist is that they are knowledgeable.
I highly give importance to the psychiatrist’s knowledge and skills.	1.000	0.392	0.459	0.474	0.634
I like to know the psychiatrist’s credentials and qualifications before meeting them.	0.392	1.000	0.686	0.385	0.330
I look into a doctor’s subspecialty before choosing them.	0.459	0.686	1.000	0.537	0.480
I want my psychiatrist to be up to date on the most recent research available.	0.474	0.385	0.537	1.000	0.575
The most important trait in a psychiatrist is that they are knowledgeable.	0.634	0.330	0.480	0.575	1.000

**Table 4:** Inter-item correlation between items in the social domain

	I give more importance to the psychiatrist’s social skills.	The psychiatrist should be clear and direct while speaking.	The psychiatrist should speak slowly and ensure my understanding.	The psychiatrist should allow me time to speak openly.	The psychiatrist should maintain eye contact at all times.	The psychiatrist should pay attention to their nonverbal communication (body language, posture, and facial expression).	I value my psychiatrist’s dress and grooming.	The most important trait in a psychiatrist is that they have good social skills.
I give more importance to the psychiatrist’s social skills.	1.000	0.664	0.635	0.665	0.554	0.490	0.541	0.684
The psychiatrist should be clear and direct while speaking.	0.664	1.000	0.730	0.767	0.542	0.569	0.529	0.699
The psychiatrist should speak slowly and ensure my understanding.	0.635	0.730	1.000	0.798	0.541	0.624	0.527	0.677
The psychiatrist should allow me time to speak openly.	0.665	0.767	0.798	1.000	0.621	0.642	0.568	0.741
The psychiatrist should maintain eye contact at all times.	0.554	0.542	0.541	0.621	1.000	0.697	0.667	0.625
The psychiatrist should pay attention to their nonverbal communication (body language, posture, and facial expression).	0.490	0.569	0.624	0.642	0.697	1.000	0.605	0.645
I value my psychiatrist’s dress and grooming.	0.541	0.529	0.527	0.568	0.667	0.605	1.000	0.682
The most important trait in a psychiatrist is that they have good social skills.	0.684	0.699	0.677	0.741	0.625	0.645	0.682	1.000

**Table 5:** Inter-item correlation between items in the clinical setting domain

	I highly give importance to the clinical setting of my psychiatrist.	I prefer physical clinics over virtual clinics (i.e., through phone, Zoom, etc.).	I prefer to speak with the psychiatrist only without my caregiver, escort, relatives, nurses, or medical interns/students.	My consultation time with the psychiatrist should be sufficient (from 15 minutes to more than one hour), and it must be valuable.	Overall, my physiatrist should be kind, knowledgeable, and skillful.
I highly give importance to the clinical setting of my psychiatrist.	1.000	0.006	0.070	0.382	0.445
I prefer physical clinics over virtual clinics (i.e., through phone, Zoom, etc.).	0.006	1.000	0.040	-0.192	-0.221
I prefer to speak with the psychiatrist only without my caregiver, escort, relatives, nurses, or medical interns/students.	0.070	0.040	1.000	0.159	0.196
My consultation time with the psychiatrist should be sufficient (from 15 minutes to more than one hour), and it must be valuable.	0.382	-0.192	0.159	1.000	0.609
Overall, my physiatrist should be kind, knowledgeable, and skillful.	0.445	-0.221	0.196	0.609	1.000

For inferential statistics, the nonparametric chi-squared test, Mann-Whitney U test, and Kruskal-Wallis test were conducted to compare preferences between respondents of different sex, marital status, qualification, occupation, controllability of disorders, etc. (Tables 6-8).

**Table 6:** Mann-Whitney U test to compare scores per item in different domains segregated by sex

Grouped by sex variable			
	Knowledge domain score per item	Social domain score per item	Social domain score per item
Mann-Whitney U	4549.500	4334.000	4815.000
p-value (two-tailed)	0.329	0.130	0.749

**Table 7:** Kruskal-Wallis test to compare scores per item in different domains segregated by demographic variables

Grouping variable	Knowledge domain score per item		Social domain score per item		Clinical setting domain score per item	
	Chi-squared	p-value	Chi-squared	p-value	Chi-squared	p-value
Marital status	1.591	0.661	4.159	0.245	1.577	0.665
Qualification	5.162	0.396	3.466	0.629	4.092	0.536
Occupation	8.974	0.110	6.561	0.255	3.214	0.667
Is the patient’s condition controllable?	9.850	0.043	6.287	0.179	3.764	0.439
Frequency of follow-up	16.338	0.006	4.849	0.435	7.419	0.191

**Table 8:** Chi-squared test to compare preferences about psychiatrist’s personal traits segregated by demographic variables

Grouping variable	Doctor’s gender		Doctor’s age	
	Chi-squared	p-value	Chi-squared	p-value
Sex	3.309	0.191	2.967	0.705
Marital status	8.827	0.184	19.377	0.197
Qualification	4.944	0.895	17.671	0.856
Occupation	8.382	0.592	43.658	0.012

Statistical analysis was carried out using Statistical Package for the Social Sciences (SPSS) (IBM SPSS Statistics, Armonk, NY, USA).

## Results

Personal demographics

In this study, 199 patients responded to the questionnaire. Among those respondents, males and females are almost equal among the patients. Except for nine, all respondents are Saudi Arabia nationals. Of those, 98.9% were from the eastern region. Furthermore, 44.7% (n=89) were married and 39.1% (n=74) hold a bachelor’s degree. Almost half of the patients are either unemployed or housewives. Finally, 68.8% (n=137) of the responses were filled by the patients themselves (Table 9).

**Table 9:** Respondents’ demographic profile

Demographic variables	Frequency (percentage) (number (%))
Sex	
Male	96 (48.24%)
Female	103 (51.75%)
Marital status	
Single	83 (41.7%)
Married	89 (44.72%)
Widowed	5 (2.51%)
Divorced	22 (11.05%)
Number of children	
1	98 (49.24%)
2	10 (5.02%)
3	22 (11.05%)
4	20 (10.05%)
5	19 (9.54%)
6	13 (6.53%)
7	3 (1.5%)
8	4 (2.01%)
9	10 (5.02%)
Qualification	
Illiterate	9 (4.52%)
Elementary/intermediate	23 (11.55%)
Highschool degree	74 (37.18%)
Bachelor’s degree	78 (39.19%)
Postgraduate	11 (5.52%)
Doctorate	4 (2.01%)
Occupation	
Unemployed/housewife	91 (45.72%)
Teacher	18 (9.04%)
Military/police	6 (3.01%)
Engineer/doctor/professor	16 (8.04%)
Retired	11 (5.52%)
Other	57 (28.64%)
Nationality	
Saudi	190 (95.47%)
Non-Saudi	9 (4.52%)
Residential area	
Eastern province	197 (98.99%)
Western region	0 (0%)
Central region	0 (0%)
Northern region	2 (1%)
Southern region	0 (0%)
Relation to patient	
Patient	137 (68.84%)
Sibling	26 (13.06%)
Parent	14 (7.03%)
Son/daughter	9 (4.52%)
Caregiver	5 (2.51%)
Other	8 (4.02%)
Continuous demographic variable: age	Mean (standard deviation)
Age	36.24 (14.166)

Patient medical data

Of the respondents, 34.17% (n=68) reported the patient’s condition as major depressive disorder, followed by 20.6% (n=41) who responded with bipolar disorder. Furthermore, 3% (n=6) reported the patient’s condition as narcolepsy disorder, 80.4% (n=160) reported the patient’s condition as controllable, and 80.4% (n=160) reported that the patient did not require hospitalization. Of the patients, 27.6% (n=55) reported that the patient generally complains about the management plan. The percentage of patients having their monthly (n=70) and quarterly (n=67) follow-ups was observed at 35% and 33%, respectively (Table 10). Also, respondents who had monthly or quarterly follow-ups scored statistically significantly higher (lesser importance) on the knowledge domain than the rest (Table 7).

**Table 10:** Patients’ medical profile

Patients’ medical variables	Frequency (percentage) (number (%))
Disorder the patient is diagnosed with	
Major depressive disorder	68 (34.17%)
Bipolar disorder	41 (20.6%)
General anxiety disorder	41 (20.6%)
Schizophrenia	24 (12.06%)
Narcolepsy	6 (3.01%)
Obsessive-compulsive disorder	14 (7.03%)
Psychotic disorder	15 (7.53%)
Dementia	7 (3.51%)
Eating disorder	5 (2.51%)
Is the patient’s condition controllable?	
Totally agree	90 (45.22%)
Agree	70 (35.17%)
Neutral	31 (15.57%)
Disagree	5 (2.51%)
Totally disagree	3 (1.5%)
Frequency of follow-up	
Weekly	16 (8.04%)
Monthly	70 (35.17%)
Quarterly	67 (33.66%)
Half-yearly	19 (9.54%)
Annually	12 (6.03%)
Other	15 (7.53%)
Did the patient ever need to be hospitalized?	
Yes	71 (35.67%)
No	128 (64.32%)
The patient generally complaints about the management plan	
Totally agree	25 (12.56%)
Agree	30 (15.07%)
Neutral	71 (35.67%)
Disagree	32 (16.08%)
Totally disagree	41 (20.6%)

Psychiatrist personal trait

While most respondents showed a clear preference for Saudi nationals and Arabic-speaking psychiatrists, more than half of the respondents said that the gender and age of the psychiatrists did not matter to them. Of the respondents, 51.7% (n=103) had no preference for the psychiatrist’s sex, 53.2% (n=107) did not have a preference regarding the psychiatrist’s age, and 59.2% (n=118) preferred Arabic-speaking psychiatrists. Conversely, 21.6% (n=43) did not have a language preference, and 85.4% (n=170) of the respondents reported that they had chosen their psychiatrist based on reputation and word of mouth (Table 11).

**Table 11:** Respondents’ preference for a psychiatrist’s personal traits

Variables	Frequency (percentage) (number (%))
Preferred gender	
Male	57 (28.64%)
Female	39 (19.59%)
No difference	103 (51.75%)
Preferred age	
30-40 years	24 (12.06%)
41-50 years	36 (18.09%)
51-60 years	32 (16.08%)
More than 60 years	5 (2.51%)
No difference	107 (53.76%)
Preferred nationality/cultural background	
Saudi	106 (53.26%)
Non-Saudi	8 (4.02%)
Does not matter	85 (42.71%)
Psychiatrist’s preferred language	
Arabic	118 (59.29%)
English	9 (4.52%)
No preference	43 (21.6%)
Only speak one language	29 (14.57%)
I chose my psychiatrist based on their reputation and word of mouth	
Totally agree	55 (27.63%)
Agree	40 (20.1%)
Neutral	75 (37.68%)
Disagree	11 (5.52%)
Totally disagree	18 (9.04%)

Appealing and unappealing attributes of a psychiatrist

Regarding the knowledge domain, 69.3% (n=138) of the respondents reported that they highly value the psychiatrist’s knowledge and skills. More than 51.7% of them wanted to know a psychiatrist’s credentials, qualifications, and subspecialty before choosing them, and 81.4% of them wanted their psychiatrist to be up to date on the most recent research. Furthermore, 91.9% reported that the most crucial trait for a psychiatrist is being knowledgeable. Further analyzing the social domain, 66.8% of the respondents reported giving more importance to the psychiatrist’s social skills, and 74.3% reported that the most critical trait for a psychiatrist is having good social skills. Of the respondents, 66.8% valued their psychiatrist’s dress and grooming, and 73.8% preferred that their psychiatrist should be clear and direct while speaking. Moreover, 77.8% reported that the psychiatrist should allow them time to speak openly. However, 48.3% reported that the psychiatrist should not always maintain eye contact. Overall, 97.7% of the respondents reported that their psychiatrist should be kind, knowledgeable, and skillful. More than 80% of the respondents either “totally agreed” or “agreed” that the psychiatrist should be up to date on the most recent research available. More than 90% of the respondents either “totally agreed” or “agreed” that being knowledgeable is a psychiatrist’s most important trait. Approximately 75% of the respondents either “totally agreed” or “agreed” that having good social skills is the most important trait of a psychiatrist. Approximately 95% of the respondents either “totally agreed” or “agreed” that their psychiatrist should be kind, knowledgeable, and skillful (Table 12).

**Table 12:** Appealing and unappealing characteristics of a psychiatrist

Questions and options for different characteristics of a psychiatrist	Frequency (percentage) (number (%))
Knowledge domain	
I highly give importance to the psychiatrist’s knowledge and skills.	
Totally agree	85 (42.71%)
Agree	53 (26.63%)
Neutral	60 (30.15%)
Disagree	0 (0%)
Totally disagree	1 (0.5%)
I like to know the psychiatrist’s credentials and qualifications before meeting them.	
Totally agree	51 (25.62%)
Agree	52 (26.13%)
Neutral	87 (43.71%)
Disagree	9 (4.52%)
Totally disagree	0 (0%)
I look into a doctor’s subspecialty before choosing them.	
Totally agree	50 (25.12%)
Agree	47 (23.61%)
Neutral	92 (46.23%)
Disagree	9 (4.52%)
Totally disagree	1 (0.5%)
I want my psychiatrist to be up to date on the most recent research available.	
Totally agree	75 (37.68%)
Agree	87 (43.71%)
Neutral	35 (17.58%)
Disagree	1 (0.5%)
Totally disagree	1 (0.5%)
The most important trait in a psychiatrist is that they are knowledgeable.	
Totally agree	102 (51.25%)
Agree	81 (40.7%)
Neutral	14 (7.03%)
Disagree	1 (0.5%)
Totally disagree	1 (0.5%)
Social domain	
I give more importance to the psychiatrist’s social skills.	
Totally agree	78 (39.19%)
Agree	55 (27.63%)
Neutral	64 (32.16%)
Disagree	2 (1%)
Totally disagree	0 (0%)
The psychiatrist should be clear and direct while speaking.	
Totally agree	93 (46.73%)
Agree	54 (27.13%)
Neutral	52 (26.13%)
Disagree	0 (0%)
Totally disagree	0 (0%)
The psychiatrist should speak slowly and ensure my understanding.	
Totally agree	89 (44.72%)
Agree	62 (31.15%)
Neutral	47 (23.61%)
Disagree	0 (0%)
Totally disagree	1 (0.5%)
The psychiatrist should allow me time to speak openly.	
Totally agree	105 (52.76%)
Agree	50 (25.12%)
Neutral	43 (21.6%)
Disagree	0 (0%)
Totally disagree	1 (0.5%)
The psychiatrist should maintain eye contact at all times.	
Totally agree	68 (34.17%)
Agree	67 (33.66%)
Neutral	62 (31.15%)
Disagree	1 (0.5%)
Totally disagree	1 (0.5%)
The psychiatrist should pay attention to their nonverbal communication (body language, posture, and facial expression).	
Totally agree	72 (36.18%)
Agree	67 (33.66%)
Neutral	55 (27.63%)
Disagree	5 (2.51%)
Totally disagree	0 (0%)
I value my psychiatrist’s dress and grooming.	
Totally agree	74 (37.18%)
Agree	59 (29.64%)
Neutral	62 (31.15%)
Disagree	3 (1.5%)
Totally disagree	1 (0.5%)
The most important trait in a psychiatrist is that they have good social skills.	
Totally agree	87 (43.71%)
Agree	61 (30.65%)
Neutral	49 (24.62%)
Disagree	2 (1%)
Totally disagree	0 (0%)
Clinical Setting Domain	
I highly give importance to the clinical setting of my psychiatrist.	
Totally agree	63 (31.65%)
Agree	56 (28.14%)
Neutral	77 (38.69%)
Disagree	3 (1.5%)
Totally disagree	0 (0%)
I prefer physical clinics over virtual clinics (i.e., through phone, Zoom, etc.).	
Totally agree	32 (16.08%)
Agree	29 (14.57%)
Neutral	45 (22.61%)
Disagree	48 (24.12%)
Totally disagree	45 (22.61%)
I prefer to speak with the psychiatrist only without my caregiver, escort, relatives, nurses, or medical interns/students.	
Totally agree	81 (40.7%)
Agree	60 (30.15%)
Neutral	47 (23.61%)
Disagree	7 (3.51%)
Totally disagree	4 (2.01%)
My consultation time with the psychiatrist should be sufficient (from 15 minutes to more than one hour), and it must be valuable.	
Totally agree	103 (51.75%)
Agree	70 (35.17%)
Neutral	22 (11.05%)
Disagree	3 (1.5%)
Totally disagree	1 (0.5%)
Overall, my physiatrist should be kind, knowledgeable, and skillful.	
Totally agree	112 (56.28%)
Agree	76 (38.19%)
Neutral	11 (5.52%)
Disagree	0 (0%)
Totally disagree	0 (0%)

The preference of psychiatrist’s traits on knowledge, social, or clinical setting domain did not statistically differ between respondents of the two genders (Table 6). No statistical difference was found in the preference for psychiatrist’s traits for respondents segregated by marital status, qualification, and occupation. However, respondents who agreed that the patient’s condition was controllable scored statistically significantly higher (lesser importance) on the knowledge domain than those who disagreed (Table 7).

## Discussion

Patients’ personal preferences toward a good and competent psychiatrist may vary depending on many different factors, including age, gender, race, religion, and much more [16]. Based on our literature review, there were no studies conducted in Saudi Arabia evaluating this topic from the patient’s perspective. For this reason, this study was conducted in Saudi Arabia to establish preferences in this region of the world.

We gathered the responses of outpatients and their guardians visiting King Fahad Hospital of the University via an online survey, breaking down different components of interest we labeled as “domains” to interpret and analyze the answers. Some of the respondents had difficulty using their electronic devices, and some had difficulty reading. Most of these respondents had a relative, caregiver, or guardian to help fill out the survey. None of the respondents complained about the inability to complete the survey. We have compared respondents’ demographics to a number of domains that can influence the decision of whether a certain psychiatrist is deemed a good and competent doctor. These domains were the personal traits, the knowledge domain, the social domain including verbal and nonverbal communication, and the clinical setting domain.

In our study, results showed that most respondents had no preference for the psychiatrist’s age. Moreover, half of the respondents had no preference regarding the physician’s sex. Furthermore, most of the respondents chose their doctor based on reputation or word of mouth. In other words, there are no obvious preferences toward a psychiatrist’s personal or demographic traits except for being Saudi and speaking in the Arabic language as they were the most favorable traits. However, more concern lies in a different domain.

The knowledge domain was highly valued by respondents as 69.34% valued their psychiatrist’s knowledge and skills. Of the patients, 81.39% preferred their psychiatrist to be up to date on the recent research available. However, the patients had a lower interest in the credentials, qualifications, and subspecialty of their psychiatrist as most of the patients’ responses were neutral (Table 12).

As for the social domain, the greatest emphasis was placed on traits such as the doctor’s rate of speech and explaining abilities. Patients preferred their psychiatrist to speak slowly and to insure their understanding. The patient-physician relationship in psychiatry is a fundamental part of the practice [17]. Results illustrate how empathy and proper communication are important for patients, as they are looking for someone who can understand them and make them feel comfortable, as well as make them understand their condition with simple words rather than complicated scientific explanations.

Although the knowledge and social domains highly influence the perception of a patient to their psychiatrist, the clinical domain was deemed the most crucial as it scored the least points per item, indicating that for the respondents, the clinical domain is the most important aspect of a psychiatrist’s practice (Table 13). The greatest emphasis in the clinical domain was on the session time where 86.92% of the respondents agreed that the consultation time with the psychiatrist should be of sufficient time from 15 minutes at least extending to more than one hour long. Moreover, having a private talk with the psychiatrist was an important need for most of the patients as 70.85% preferred to speak with the psychiatrists only without their caregiver, escort, relatives, nurses, or medical students. Many patients preferred virtual clinics over physical clinics (Table 12). Managing professional boundaries and principles, ensuring privacy and confidentiality, and creating realistic expectations regarding digital interactions are key ethical issues that digital technology creates for psychiatrists [18].

**Table 13:** Mean and standard deviation for the calculated scores per item for each of the knowledge, social, and clinical setting domain

Variable	Mean	Standard deviation
Score per item in the knowledge domain	2.437	0.7264
Score per item in the social domain	2.146	0.8008
Score per item in the clinical setting domain	2.083	0.4933

Our results suggest that the clinical domain is an integral part of a psychiatrist’s practice as these settings allow the psychiatrist to openly discuss treatment with the patient in a respectful and understanding environment, ensuring privacy and enough time for them to discuss their complaints with comfort [19]. Incorporation of these adjustments and skills from different domains in the approach to teaching and treating mental health might improve the overall outcome and patient satisfaction.

Of the respondents, 95% were Arabic speakers and 60% preferred their psychiatrist to speak in Arabic. Therefore, we recommend that psychiatrists must be made aware of patients’ preferences for language and refrain from using other languages in the interview. If the psychiatrist needs to speak in another language in the context of explaining the disorder or the management plan, the patient should be informed and offered the option of his/her preferred language.

Furthermore, 68% of the patients reported that physical clinics have no advantage over virtual clinics. Therefore, we recommend that virtual clinics be incorporated into the psychiatry practice and for patients be given the choice of virtual or physical clinics. This in turn will decrease the patient load in the hospital and improve access to healthcare for patients in more remote places.

Moreover, 70% of the patients prefer to speak to the psychiatrist alone. This was not a surprising result; as we know, the psychiatrist-patient relationship especially can be sensitive and private to the patients more than any other specialty. In teaching hospitals such as KFHU, the outpatient department is a valuable teaching setting for interns and students. However, there must be a balance between teaching and patient care. There is a constant dilemma in teaching hospitals of balancing teaching opportunities and patient privacy. Based on patient preferences in our region, we suggest that consent for students’ and interns’ attendance at clinics be taken with care. Our research focuses on the patient’s perspective; however, the impact of our recommendation on the teaching capacity and competence of trainees is not well studied in our research.

Of the patients, 50% prefer to know the psychiatrist’s credentials, qualifications, and subspecialty before choosing or meeting them. Therefore, making the psychiatrist’s credentials and qualifications readily accessible and clear to the patients, whether on the hospital website or made available in the outpatient department, can improve patient satisfaction and willingness to continue following up. Although only 5% of the patients preferred not knowing the credentials and qualifications of the psychiatrist, we believe implementing these changes has an overall positive effect on the psychiatrist’s practice.

In a qualitative study conducted on Singaporean psychiatrists, patients were especially concerned about the social characteristics of the psychiatrist. Extra efforts from the psychiatrist were a key factor in convincing patients to go through the management plan, as it represented care and sympathy. Patients preferred physicians from an opposite race and gender as they were looking for a more open-minded, nonjudgmental physician that can understand them without judging them [10].

A study conducted in the UK found that the most appreciated characteristic of a competent psychiatrist was their ability to make sound clinical decisions, followed by the ability to appraise staff members. This indicates that the participants were less concerned with the organizational social aspects of the medical processes than the clinical management components [20].

Although the overall requirement for a knowledgeable, skilled, and socially competent psychiatrist and a proper clinical setting is sought by most psychiatric patients in different cultures and countries, there are also significant differences in the priorities of such characteristics. This emphasizes how every region has its standards for this occupation [21,22].

Limitations and recommendations

Having a relatively low response rate of approximately 25%, not having any respondents from the inpatient department, and not taking into consideration the non-Islamic religions are the limitations of the study. The results of this study are not applicable to inpatient departments or non-Muslims. We recommend for future studies of this nature to target a larger sample size and include different regions of Saudi Arabia to have a broader appreciation of differences within the country. Also, more research of such kind is needed in different countries, cultures, and religious groups. Moreover, research examining the relationship between the quality of medical teaching outcomes and patient privacy is needed in our region.

## Conclusions

In conclusion, our results showed that the clinical setting was the most critical domain, followed by the social and knowledge domains. To improve patient experience and satisfaction, certain actions should be taken into consideration in our region. The patient should be made aware of the psychiatrist’s language and offered the option of their preferred language. Moreover, incorporating virtual clinics into the psychiatry practice has benefits for both the hospital and the patients. Furthermore, psychiatrists in teaching hospitals should take consent from the patient regarding the attendance of students and interns in the clinic. Finally, the psychiatrist’s credentials, qualifications, and subspecialty should be readily available and clear to all patients. These results suggest a variety in patients’ and guardians’ perspectives in different cultures with the clinical setting being an integral part of psychiatrist’s practice in our region.
